# Association between pre-diagnostic prevalence of 30 common diseases and the subsequent risk of dementia: a population-based retrospective cohort study in Taiwan

**DOI:** 10.3389/fnagi.2026.1784183

**Published:** 2026-04-17

**Authors:** Yibo Li, Lei Qin, Yi-Wei Kao, Yiling Zhang, Qi Zhu, Di Deng, Yuejian Zhang, Ben-Chang Shia, Tao Lu, Ping Wen

**Affiliations:** 1 Shenzhen Maternity and Child Healthcare Hospital, Women and Children’s Medical Center, Southern Medical University, Shenzhen, China; 2 School of Life Sciences, Beijing University of Chinese Medicine, Beijing, China; 3 School of Statistics, University of International Business and Economics, Beijing, China; 4 Graduate Institute of Business Administration, Fu Jen Catholic University, Taipei, Taiwan; 5 Xiamen Municipal Health Commission, Tong An Lu 2 Hao, Xiamen, Fujian, China

**Keywords:** clinical profile, comorbidity, correlates, dementia, epidemiology

## Abstract

**Background:**

Dementia associates with long-term progression, but the health status of individuals years before diagnosis remains poorly understood. We therefore conducted a large retrospective cohort study to examine the association between early-life morbidity and incident dementia.

**Methods:**

We designed a large-scale retrospective matched-cohort study using data from the National Health Insurance Research Database of Taiwan. Individuals newly diagnosed with dementia (2010–2013) were identified as the pre-dementia cohort and matched 1:3 with non-dementia controls. We assessed prevalence and outpatient visits for 30 common conditions (infectious to various of cancers.) between 2000 and 2013, with a backtracking time of 10 years for each individual. Conditional logistic regression was used to estimate odds ratios (ORs) for disease prevalence, while Negative Binomial regression was applied to calculate Incidence Rate Ratios (IRRs) for outpatient visit.

**Results:**

From 806,292 individuals, 19 of 30 diseases showed significant associations. Notably, the pre-dementia cohort exhibited a significantly lower prevalence of and fewer hospital visits for 13 conditions compared to controls. These included acute respiratory infections, peripheral inflammatory diseases, and certain digestive system malignancies. Conversely, a significantly higher prevalence was observed in only six conditions, predominantly those associated with direct or indirect brain injury, as well as cancers of the respiratory and urinary systems.

**Conclusion:**

Pre-dementia patients showed lower prevalence of most correlated common diseases. These unexpected findings are consistent with the hypothesis that altered peripheral immune function may be involved in the etiology and pathogenesis of dementia, which is beneficial for normal health but detrimental to the brain. Meanwhile, conditions that directly or indirectly contribute to brain damage are more likely to be positively associated with dementia. However, given the observational nature of our study and the absence of immunological biomarkers, these mechanistic interpretations remain speculative and require further validation.

## Background

Dementia is a grave and prevailing neurodegenerative disease (Prince et al., 2013), characterized by progressive cognitive decline, functional deterioration, and increased mortality (Sommerlad et al., 2023). The etiology and pathogenesis of dementia has been a subject of great interest but remained largely unclear.

Correlational studies for comorbidities have been a constructive means to provide information on the etiology and pathogenesis of dementia, but are rather delicate to design. Also, it is hard to rule out the effects from dementia related medications; This is, however, not the case for retrospective studies, which inspect the situation years before the dementia diagnosis.

A 60–70% of dementia cases are caused by Alzheimer’s disease (AD) (Wortmann, 2012), and there is a strong link between cerebrovascular damage, such as traumatic brain injury (TBI), and the development of dementia (Graham and Sharp, 2023; Mok et al., 2024), but other non-brain damage relationships are not entirely understood. Growing evidence suggests that systemic infections and peripheral inflammations are also related to dementia (Gao et al., 2023; Janbek et al., 2023; Zhang et al., 2023). One large-scale cohort study reported that patients with gout have a lower risk of developing AD (Lu et al., 2016); while an inverse association between cancer and dementia was also found in other studies (Musicco et al., 2013; Shafi, 2016). Despite these reports, little is known about the morbidity of early diseases among patients who later develop dementia (Patients who were diagnosed with mild cognitive impairment or other early cognitive decline markers were identified, hereafter referred to as the pre-dementia group). To identify differences in the morbidity of pre-dementia patients from those non-dementia cohort might shed light on the etiology and development of dementia.

Based on previous findings, we hypothesized that the decade preceding dementia diagnosis represents a critical window reflecting distinct peripheral and central inflammatory responses. Utilizing the National Health Insurance Research Database (NHIRD) of Taiwan, we conducted a large-scale, population-based retrospective matched-cohort study. The primary objectives were to (1) explore the relationship between pre-dementia and the morbidity of diseases, including common infectious diseases, inflammatory conditions, as well as multiple types of cancer; (2) generate hypotheses regarding the potential etiology and pathology of dementia based on observed disease association patterns.

## Materials and methods

### Study design and participants

**FIGURE 1:**
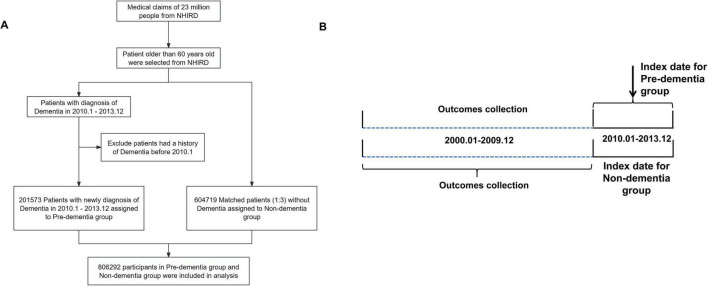
**(A)** Flow diagram. **(B)** Outcomes selection. Data were extracted from the National Health Insurance Research Database (NHIRD) of Taiwan from 2000 to 2013. From this database, 201, 573 were identified as patients newly diagnosed with dementia from Jan. 2010 to Dec. 2013. A total of 604, 719 control patients without a diagnosis of dementia were matched by propensity score pertaining to baseline characteristics (age, sex, income, region and urbanization level) and certain comorbidities (hypertension, hyperlipidemia and diabetes). Total 806, 292 participants were selected in this cohort study.

This retrospective matched-cohort study enrolled participants aged 60 years or older with data spanning from 1 January 2000 to 31 December 2013. Patients newly diagnosed with dementia between January 1, 2010, and December 31, 2013, were identified as the pre-dementia cohort, and the date of their first dementia diagnosis was defined as the index date. A non-dementia control cohort was subsequently established by matching individuals to the pre-dementia cohort at a ratio of 1:3 on age, sex, and baseline characteristics. Importantly, each matched control was assigned the same index date as the corresponding dementia case to ensure aligned observation windows and temporal symmetry in the 10-year look-back analysis. Individuals with any diagnosis of dementia before or on the index date were excluded from the control group. Both cohorts were subsequently evaluated for the prevalence of 30 common diseases and associated hospital visits within the decade preceding their respective index dates (Figure 1).

### Ethical approval

The study protocol was approved by the Regional Ethics Review Board of Taipei Medical University, Taiwan (TMU-JIRB No. N201712044). All patient data were de-identified prior to analysis. No industry funding or support was received for this study.

### Data sources

Data were extracted from the National Health Insurance Research Database (NHIRD) of Taiwan, a comprehensive database encompassing approximately 23 million individuals, representing the entire Taiwanese population, for the period from 2000 to 2013. The dataset included all outpatient visit records, comprising demographic details, socioeconomic variables, and both past and present diagnoses. No manual data abstraction was performed.

### Outcomes measures and disease definition

We assessed the 10-year prevalence of 30 predefined common diseases and associated outpatient hospital visits in both cohorts prior to their respective index dates. All disease diagnoses, including our primary outcome, were identified from outpatient records using International Classification of Diseases, Ninth Revision, Clinical Modification (ICD-9-CM) codes. Dementia was defined as all-cause dementia based on ICD-9-CM codes is provided in Supplementary Table 1. Because administrative coding in routine clinical practice has inherent limitations in reliably distinguishing dementia subtypes, our analysis was not restricted to any specific subtype. This approach is consistent with that used in other large-scale epidemiological studies based on administrative healthcare databases (Blass et al., 2025; Carcaillon-Bentata et al., 2021).

The 30 common diseases evaluated included: acute nasopharyngitis (common cold), acute sinusitis, pharyngitis, acute tonsillitis, acute laryngitis and tracheitis, acute bronchitis and bronchiolitis, gastritis and duodenitis, acute appendicitis, urticaria, blepharitis, contusion of lower limb, gout, occlusion of cerebral arteries (OCA), and 17 types of cancer. The cancers investigated were: head and neck cancer, brain cancer, lung cancer, esophagus cancer, pancreas cancer, stomach cancer, small intestine cancer, colon cancer, liver cancer, uterine cancer, prostate cancer, bladder cancer, kidney cancer, skin cancer, breast cancer, thyroid cancer, and hematologic diseases.

### Statistical analysis

Descriptive analyses were performed to characterize both cohorts. Propensity-score methods were used to balance baseline characteristics, including age group (60–64, 65–69, 70–74, 75–79, ≥ 80), gender (male, female), monthly income (NT$0–15840, NT$15841–25000, ≥ NT$25001), geographical location (northern, central, eastern, and southern Taiwan), urbanization level and pre-existing comorbidities (hypertension, hyperlipidemia and diabetes). Chi-squared tests were employed to compare the distribution of categorical variables between the two cohorts.

Because we evaluated associations with 30 distinct clinical outcomes, we applied the Benjamini-Hochberg False Discovery Rate (FDR) procedure to correct for multiple testing. Associations were considered statistically significant if the FDR-adjusted *P*-value (*q*-value) was < 0.05.

The prevalence of the 30 common diseases within the target 10-year period was measured for both cohorts. Conditional logistic regression models were utilized to calculate crude odds ratios (ORs) and subsequent multivariate-adjusted ORs, accounting for all aforementioned baseline characteristics. Results are presented as the number of patients with their corresponding percentage, and adjusted ORs with 95% confidence intervals (95% CIs) for each cohort. To further evaluate healthcare utilization patterns, we analyzed the frequency of hospital visits related to the target diseases in both cohorts. Given that hospital visit counts were inherently right-skewed and exhibited significant overdispersion (with variances substantially exceeding the means), we employed Negative Binomial (NB) regression models to compare the visit frequencies between the pre-dementia and non-dementia groups. The results are presented as Incidence Rate Ratios (IRRs) with their corresponding 95% confidence intervals (CIs). Descriptive statistics are provided as means to facilitate clinical interpretation, while the statistical significance of the differences was rigorously determined by the NB models.

All statistical analyses were conducted with SAS software on Windows System (version 9.2, SAS Institute, Cary, NC). Statistical significance was set at *P*-value less than 0.05.

## Results

### Cohort characteristics

A total of 201,573 patients were identified as the pre-dementia group, while another 604,719 patients without dementia were matched and included as the controlled non-dementia group (Figure 1). Baseline demographic characteristics and medical comorbidities for both cohorts are presented in Table 1. No statistically significant differences were observed between the two cohorts regarding sex, monthly insured income, geographical region, urbanization level, hypertension, or diabetes. Although small statistically significant differences remained in mean age (77.86 vs. 77.80 years, *P* = 0.009) and hyperlipidemia prevalence (38.0% vs. 38.9%, *P* < 0.001) after matching, these differences represent minimal absolute differences (0.06 years and 0.9 percentage points, respectively) that are unlikely to be clinically meaningful given the large sample size.

**TABLE 1:** Baseline characteristics.

Characteristic	Pre-dementia (*n* = 201,573)	Non-dementia (*n* = 604,719)	*P*-value for difference
Age, mean (SD), y	77.86 (8.14)	77.80 (8.23)	0.009
Age (years group)			0.001
60–64	12,910 (6.4)	40,063 (6.6)	
65–69	22,000 (10.9)	66,984 (11.1)	
70–74	34,361 (17.0)	102,182 (16.9)	
75–79	41,792 (20.7)	125,488 (20.8)	
≥ 80	90,510 (44.9)	270,002 (44.6)	
Sex			0.59
Male	91,132 (45.2)	272,972 (45.1)	
Female	110,441 (54.8)	331,747(54.9)	
Monthly income			0.97
≤ NT$15,840	71,894 (35.7)	215,775 (35.7)	
NT$15,841–25,000	89,975 (44.6)	269,973 (44.6)	
≥ NT$25,001	39,704 (19.7)	118,971 (19.7)	
Geographical region			0.96
Northern	85,537 (42.4)	256,482 (42.4)	
Central	53,191 (26.4)	159,469 (26.4)	
Southern	56,448 (28.0)	169,663 (28.1)	
Eastern	6,397 (3.2)	19,105 (3.2)	
Urbanization level (mostly urbanized)	69,380 (34.4)	208,343 (34.5)	0.91
Comorbidity			
Hypertension	154,655 (76.7)	464,280 (76.8)	0.63
Hyperlipidemia	76,688 (38.0)	235,335 (38.9)	<0.001
Diabetes	79,911 (39.6)	239,477 (39.6)	0.74

The non-dementia control cohort was matched for age, sex, income, region, and urbanization level as well as for certain comorbidities (hypertension and diabetes), based on data from the National Health Insurance Research Database (NHIRD) of Taiwan. All information regarding patients with dementia was obtained from the NHIRD of Taiwan. All data used was de-identified secondary data, with no data left out. Data are presented as number (percentage) of patients unless otherwise indicated.

### Prevalence of acute respiratory infections

**FIGURE 2:**
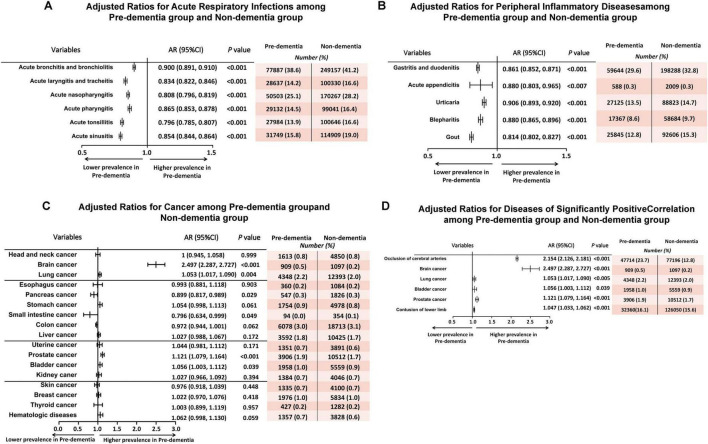
Adjusted ratios for outcomes in acute respiratory infections **(A)**, peripheral inflammatory diseases **(B)**, cancer **(C)**, diseases of significantly positive correlation **(D)**, among two cohorts. Adjusted ratios show the prevalence of each outcome among patients with dementia, as compared with matched controls. These two cohorts were evaluated retrospectively for diagnoses of 30 common diseases within a decade (Jan. 2000–Dec. 2009) prior to the diagnosis of dementia. Adjusted Ratios (AR) < 1 indicate a lower prevalence in the pre-dementia group compared to the non-dementia group. AR, Adjusted Ratio; CI, Confidence Interval.

The pre-dementia cohort exhibited a consistently lower prevalence of acute respiratory infections (Figure 2A). Specifically, the adjusted odds ratios (ORs) for acute bronchitis and bronchiolitis [0.900 (95% CI, 0.891–0.910), *P* < 0.001], acute laryngitis and tracheitis [0.834 (95% CI, 0.822–0.846), *P* < 0.001], acute nasopharyngitis [0.808 (95% CI, 0.796–0.819), *P* < 0.001], acute pharyngitis [0.865 (95% CI, 0.853–0.878), *P* < 0.001], acute tonsillitis [0.796 (95% CI, 0.785–0.807), *P* < 0.001] and acute sinusitis [0.854 (95% CI, 0.844–0.864), *P* < 0.001] were significantly lower in the pre-dementia group compared to the non-dementia group.

**FIGURE 3:**
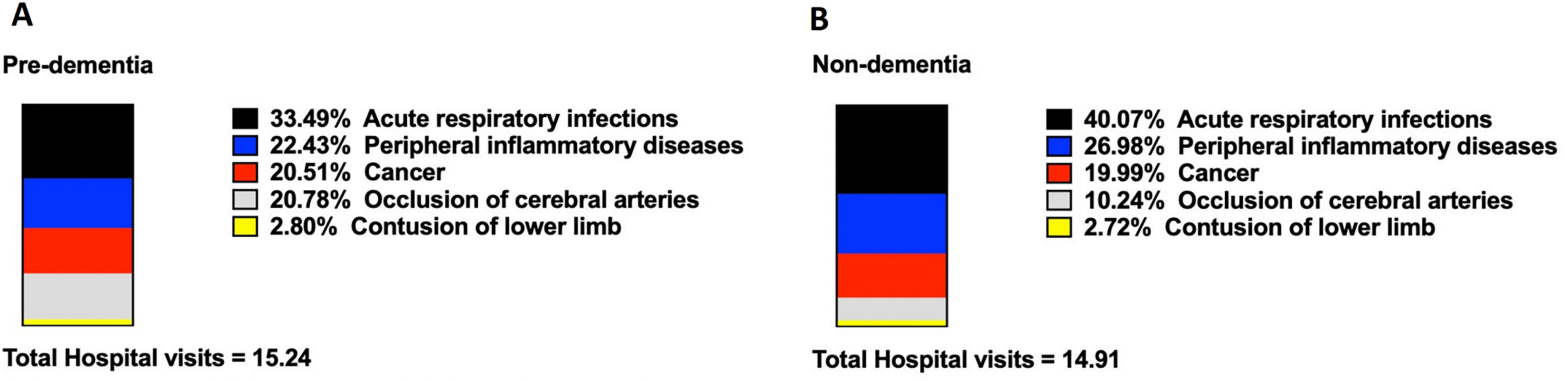
The percentage of hospital visits of diseases in pre-dementia cohort **(A)** and Non-dementia cohort **(B)**. Hospital visits were identified in hospital discharge records with use of codes in the International Classification of Diseases, Ninth Revision. Acute Respiratory Infections include acute bronchitis and bronchiolitis, acute laryngitis and tracheitis, acute tonsilitis, acute pharyngitis, acute sinusitis and acute nasopharyngitis. Peripheral Inflammatory Diseases include gastritis and duodenitis, urticaria, acute appendicitis and blepharitis. Cancer includes 17 types of cancer. These two groups were evaluated retrospectively for hospital visits of the diseases within a decade (Jan. 2000–Dec. 2009) prior to the diagnosis of dementia. All the data was de-identified secondary data. No missing data.

Consistent with these prevalence findings, the pre-dementia cohort also demonstrated significantly lower rates of hospital visits for acute bronchitis and bronchiolitis (IRR = 0.86, 95% CI: 0.85–0.87, FDR-adjusted *P* < 0.003), acute laryngitis and tracheitis (IRR = 0.82, 95% CI: 0.80–0.84, FDR-adjusted *P* < 0.003), acute tonsillitis (IRR = 0.83, 95% CI: 0.80–0.85, FDR-adjusted *P* < 0.003), acute pharyngitis (IRR = 0.84, 95% CI: 0.82–0.86, FDR-adjusted *P* < 0.003), acute sinusitis (IRR = 0.80, 95% CI: 0.78–0.82, FDR-adjusted *P* < 0.003) and acute nasopharyngitis (IRR = 0.82, 95% CI: 0.80–0.83, FDR-adjusted *P* < 0.003) (Table 2). Overall, the percentage of total hospital visits for acute respiratory infections was 6.58% lower in the pre-dementia group (33.49% vs. 40.07%, pre-dementia group vs. non-dementia group, Figure 3).

**TABLE 2:** Hospital visits.

Diseases	Pre-dementia (*n* = 201,573)	Non-dementia (*n* = 604,719)	IRR (95% CI)	*P*-value for difference	FDR-adjusted *P*-value
	Mean (SD)				
Acute bronchitis and bronchiolitis	1.99 (5.85)	2.32 (6.61)	0.86 (0.85, 0.87)	< 0.001	<0.003
Acute laryngitis and tracheitis	0.58 (2.99)	0.71 (3.43)	0.82 (0.80, 0.84)	< 0.001	<0.003
Acute tonsillitis	0.47 (2.43)	0.57 (2.85)	0.83 (0.80, 0.85)	< 0.001	<0.003
Acute pharyngitis	0.46 (2.32)	0.55 (2.63)	0.84 (0.82, 0.86)	< 0.001	<0.003
Acute sinusitis	0.67 (3.26)	0.84 (3.63)	0.80 (0.78, 0.82)	< 0.001	<0.003
Acute nasopharyngitis	1.10 (4.13)	1.35 (4.90)	0.82 (0.80, 0.83)	< 0.001	<0.003
Gastritis and duodenitis	1.68 (5.96)	1.97 (6.71)	0.85 (0.84, 0.87)	< 0.001	<0.003
Acute appendicitis	0.01 (0.25)	0.01 (0.22)	1.00 (0.89, 1.13)	0.51	0.612
Urticaria	0.56 (3.01)	0.64 (3.39)	0.88 (0.85, 0.90)	< 0.001	<0.003
Blepharitis	0.29 (1.77)	0.35 (2.17)	0.83 (0.80, 0.86)	< 0.001	<0.003
Gout	0.99 (4.72)	1.30 (5.47)	0.76 (0.74, 0.78)	< 0.001	<0.003
Head and neck cancer	0.19 (3.51)	0.19 (3.37)	1.00 (0.91, 1.10)	0.99	0.99
Brain cancer	0.06 (1.78)	0.02 (0.88)	3.00 (2.53, 3.56)	< 0.001	<0.003
Lung cancer	0.35 (4.20)	0.36 (4.32)	0.97 (0.92, 1.03)	0.24	0.360
Esophagus cancer	0.03 (1.18)	0.03 (1.26)	1.00 (0.82, 1.23)	0.17	0.268
Pancreas cancer	0.028 (1.01)	0.034 (1.09)	0.82 (0.69, 0.98)	< 0.005	0.010
Stomach cancer	0.16 (2.80)	0.15 (2.74)	1.07 (0.98, 1.17)	0.79	0.912
Small intestine cancer	0.01 (0.57)	0.01 (0.56)	1.00 (0.75, 1.33)	0.45	0.563
Colon cancer	0.57 (5.45)	0.60 (5.31)	0.95 (0.91, 1.00)	0.03	0.056
Liver cancer	0.33 (3.93)	0.32 (3.91)	1.03 (0.97, 1.10)	0.89	0.954
Uterine cancer	0.13 (2.60)	0.13 (2.63)	1.00 (0.90, 1.11)	0.88	0.954
Prostate cancer	0.47 (5.02)	0.43 (4.68)	1.09 (1.04, 1.15)	< 0.003	0.006
Bladder cancer	0.22 (3.47)	0.21 (3.21)	1.05 (0.97, 1.13)	0.44	0.563
Kidney cancer	0.13 (2.68)	0.13 (2.44)	1.00 (0.91, 1.10)	0.38	0.518
Skin cancer	0.11 (0.83)	0.13 (0.90)	0.85 (0.82, 0.88)	< 0.001	<0.003
Breast cancer	0.25 (3.86)	0.24 (3.59)	1.04 (0.97, 1.12)	0.35	0.5
Thyroid cancer	0.05 (1.69)	0.05 (1.57)	1.00 (0.85, 1.18)	0.97	0.99
Hematologic diseases	0.14 (2.70)	0.13 (2.70)	1.08 (0.97, 1.19)	0.10	0.176
Occlusion of cerebral arteries	3.27 (12.16)	1.62 (8.08)	2.02 (1.98, 2.06)	< 0.001	<0.003
Contusion of lower limb	0.44 (2.02)	0.43 (1.92)	1.02 (0.99, 1.06)	0.12	0.2

Hospital visits of both cohorts associated with different diseases from 01/01/2000 to 12/31/2013. The outcomes for both cohorts were collected for a timespan starting a decade before the relative index dates. The index date for members of the pre-dementia group was the exact date of diagnosis of dementia (from 01/01/2010 to 12/31/2013), each matched control was assigned the same index date as the corresponding dementia case to ensure aligned observation windows and temporal symmetry in the 10-year look-back analysis. All information was obtained from the National Health Insurance Research Database (NHIRD) of Taiwan. All data used was de-identified secondary data with no data left out. Data are presented as mean (SD).

### Prevalence of peripheral inflammatory diseases

Analysis of other peripheral inflammatory diseases revealed similar patterns (Figure 2B). The pre-dementia cohort had a significantly lower adjusted OR for gastritis and duodenitis [0.861 (95% CI, 0.852–0.871), *P* < 0.001], acute appendicitis [0.880 (95% CI, 0.803–0.965), *P* < 0.007], urticaria [0.906 (95% CI, 0.893–0.920), *P* < 0.001], blepharitis [0.880 (95% CI, 0.865–0.896), *P* < 0.001] and gout [0.814 (95% CI, 0.802–0.827), *P* < 0.001].

Correspondingly, fewer hospital visits were associated with these peripheral inflammatory diseases in the pre-dementia group (Table 2). Significant reductions were observed for gastritis and duodenitis (IRR = 0.85, 95% CI: 0.84–0.87, FDR-adjusted *P* < 0.003), urticaria (IRR = 0.88, 95% CI: 0.85–0.90, FDR-adjusted *P* < 0.003), blepharitis (IRR = 0.83, 95% CI: 0.80–0.86, FDR-adjusted *P* < 0.003) and gout (IRR = 0.76, 95% CI: 0.74–0.78, FDR-adjusted *P* < 0.003). However, hospital visits for acute appendicitis did not show a significant difference between the groups (IRR = 1.00, 95% CI: 0.89–1.13, FDR-adjusted *P* = 0.612). The overall percentage of total hospital visits related to these peripheral inflammatory diseases was lower in the pre-dementia group (22.43% vs. 26.98%, Figure 3).

### Prevalence of cancer

Among the 17 types of cancer analyzed, six demonstrated significant correlations with the pre-dementia status (Figure 2C). The pre-dementia cohort exhibited a significantly lower adjusted OR for pancreas cancer [0.899 (95% CI, 0.817–0.989), *P* = 0.029] and small intestine cancer [0.796 (95% CI, 0.634–0.999), *P* = 0.049].

Conversely, a positive correlation was observed for cancers typically associated with brain damage, the respiratory, and urinary systems. Patients in the pre-dementia group had significantly higher adjusted ORs for brain cancer [2.497 (95% CI, 2.287–2.727), *P* < 0.001], lung cancer [1.053 (95% CI, 1.017–1.090), *P* = 0.004], prostate cancer [1.121 (95% CI, 1.079–1.164), *P* < 0.001], and bladder cancer [1.056 (95% CI, 1.003–1.112), *P* = 0.039] compared to the non-dementia group.

Hospital visit trends for cancer largely mirrored these prevalence findings (Table 2). The pre-dementia group had fewer hospital visits related to pancreas cancer (IRR = 0.82, 95% CI: 0.69–0.98, FDR-adjusted *P* = 0.010), and showed a trend toward fewer visits for colon cancer (IRR = 0.95, 95% CI: 0.91–1.00, FDR-adjusted *P* = 0.056), though it did not reach statistical significance. Conversely, hospital visits for prostate cancer (IRR = 1.09, 95% CI: 1.04–1.15, FDR-adjusted *P* = 0.006) and brain cancer (IRR = 3.00, 95% CI: 2.53–3.56, FDR-adjusted *P* < 0.003) were significantly higher in the pre-dementia group.

### Diseases with significantly positive correlation

Among the analysis of all 30 common diseases, we noticed that 6 types of disease showed a significant higher prevalence in the pre-dementia cohort (Figure 2D). These included OCA [2.154 (95% CI, 2.126–2.181), *P* < 0.001], contusion of lower limb [1.047 (95% CI, 1.033–1.062), *P* < 0.001] and four cancer types previously mentioned (brain, lung, prostate, and bladder cancer). Hospital visits associated with OCA were also markedly higher in the pre-dementia group (IRR = 2.02, 95% CI: 1.98–2.06, FDR-adjusted *P* < 0.003) compared to the non-dementia group.

## Discussion

Our analysis of the NHIRD from 2000 to 2013 showed that pre-dementia patients generally had a lower recorded prevalence of, and fewer healthcare visits for, infectious diseases compared to matched non-dementia controls. Out of the 30 common diseases studied, there are certain trends within 11 kinds of disease, but not statistically significant, while 19 others were found to be significantly correlated. Surprisingly, among these 19 associated diseases, individuals in pre-dementia group had a significantly lower prevalence of 13 kinds of disease, especially in all the acute respiratory infections and peripheral inflammatory diseases studied. Only 6 out of 30 showed a significantly higher prevalence in the pre-dementia cohort, most of the diseases related to direct or indirect damage to the brain including OCA, brain cancer and lung cancer, etc. These findings provide intriguing evidence of a lower recorded incidence of certain conditions among individuals in the pre-dementia phase compared with cognitively normal controls; however, this pattern is more likely to reflect differences in healthcare utilization and disease ascertainment than a genuinely healthier overall physiological state. The possibility of altered peripheral immune status warrants further investigation.

A critical limitation of administrative claims data is surveillance bias driven by differential healthcare utilization, which plays a dual role in our study. On one hand, it may act as a mediator on the causal pathway, as individuals in the prodromal phase of dementia might experience non-specific early symptoms leading to more frequent medical encounters, thereby inflating the likelihood of diagnosing other comorbidities. On the other hand, cognitive decline can fundamentally compromise care-seeking behavior (e.g., missed appointments, communication barriers, treatment non-adherence) (Couret et al., 2024). To mitigate these effects and avoid post-diagnosis confounders, we implemented a 10-year pre-diagnosis window, adjusted for healthcare access proxies, and analyzed outpatient trajectories across 30 disease categories. This pre-diagnosis focus partially mitigates bias driven by severe cognitive decline and provides clearer insight into our findings: the observed pattern of fewer visits for many common conditions but increased visits for neurological and brain disorders among future dementia cases. Nevertheless, we acknowledge that some residual surveillance bias may persist. The observed associations for conditions typically detected through routine primary care might still be more susceptible to overestimation or underestimation than those for acute, severe conditions with distinct clinical presentations. Future studies utilizing prospectively standardized clinical assessments are required to fully disentangle the complex relationship between medical surveillance, comorbidity burden, and dementia onset.

Our study used all-cause dementia as the primary outcome, which included Alzheimer’s disease, vascular dementia, and other dementia subtypes. We acknowledge this as a limitation, as emerging evidence indicates that the association between systemic conditions (including cancer) and dementia may be subtype-specific. Recent studies suggest that certain cancer types may predominantly reduce the risk of AD rather than vascular dementia (Ma et al., 2025), with potential protective mechanisms involving specific alterations in amyloid-beta pathology (Li et al., 2026). Given that an estimated 60–70% of the dementia cases in our study cohort are likely attributable to AD, our findings may be primarily driven by AD-related pathology. However, we were unable to determine with certainty whether the observed inverse association between the pre-dementia state and infectious/inflammatory diseases applies equally across all dementia subtypes. Future studies with detailed clinical phenotyping and biomarker-based confirmation of dementia subtypes are needed to address this important issue.

While our propensity-score matching accounted for the presence of hyperlipidemia as a binary comorbidity, we acknowledge that we did not capture the age at diagnosis or duration of hyperlipidemia. As recently demonstrated by Pan et al. (2024), younger age at hyperlipidemia diagnosis is associated with increased dementia risk through a dose-response relationship. The small residual imbalance in hyperlipidemia prevalence (38.0% vs. 38.9%) in our study may therefore have a minimal influence on our observed associations. Given that both cohorts were matched on hyperlipidemia status and the absolute difference was less than 1 percentage point, any residual confounding effect is likely negligible. However, we cannot completely exclude the possibility of residual confounding by age and hyperlipidemia status. Future studies incorporating detailed lipid profiles and duration of exposure are warranted to further elucidate this relationship.

The observed reduced prevalence of acute infectious and peripheral inflammatory diseases in the decade preceding dementia diagnosis is particularly noteworthy. This finding aligns with the growing hypothesis that amyloid-beta (Aβ) (Lu et al., 2014; Minter et al., 2016), a hallmark protein in AD may function as an antimicrobial peptide, playing a protective role against infections (Ferreiro et al., 2023; Ingelsson et al., 2004). Recent reviews suggest that Aβ might serve as a part of the innate immune response against infections (Abbott, 2020; Welling et al., 2015), and therefore, it plays a protective role for the central nervous system (CNS) before at some point reversing its effect and becoming detrimental (Chen and Holtzman, 2022). Studies have suggested that pre-dementia correlates with the activation of peripheral immunity (Ashraf et al., 2019; Magalhães et al., 2018; Su et al., 2019). A shift of early to activated CD4 + T-cells in AD patients was reported (Joshi et al., 2022). Meanwhile, the adaptive immune system in AD patients was found to be in a more activated state than those of the controls. Furthermore, increased levels of pro-inflammatory factors, such as tumor necrosis factor alpha (TNF-α) (Wang et al., 2023), were found in the serum of patients with AD, indicating the presence of systemic peripheral inflammation in AD patients (Holmes et al., 2009; Magalhães et al., 2018).

The complex interaction between the peripheral immune system and the CNS is increasingly recognized (Mateus et al., 2020). While the immune system serves a dual role in both protecting against and contributing to disease (El Kadmiri et al., 2018; Magalhães et al., 2018), excessive or long-term activation of the peripheral immune response may lead to persistent neuroinflammation. For instance, macrophages in the peripheral nervous system (PNS) exhibit both pro-inflammatory (M1) and anti-inflammatory (M2) phenotypes (Huang et al., 2020). Similarly, the innate microglia in the CNS initially phagocytose debris and Aβ fibrils, exerting a protective effect (Baligács et al., 2024), but in the case of long-term activation, they may transform into neurotoxic (M1) phenotype, thereby triggering disease pathological processes (Calsolaro and Edison, 2016; Fan et al., 2017). Emerging research further indicates that viral infections such as herpes simplex virus type 1 are associated with a reduced infection rate due to Aβ deposition (Eimer et al., 2018; Powell-Doherty et al., 2020), and links innate immune proteins in modulating Aβ production upon viral entry into the brain, thereby increasing AD risk (Hur et al., 2020; Rathore et al., 2018). Our results, showing lower incidence rates of acute infectious and inflammatory diseases in the pre-dementia cohort, are consistent with the hypothesis that peripheral immune activation may contribute to elevated Aβ and inflammatory factors, thereby potentially promoting AD development. However, this mechanistic interpretation remains speculative and requires validation in future studies with immunological biomarkers. This is further supported by the observation that long-term use of non-steroidal anti-inflammatory drugs (NSAIDs) may offer protection against AD (Etminan et al., 2003; Jordan et al., 2020), suggesting a role for inflammation modulation.

Cancer and AD are both associated with inflammation and aging. Epidemiological studies and systematic reviews have demonstrated a robust reciprocal association between cancer and AD, indicating that elderly individuals with AD or dementia have a decreased risk of developing cancer and vice versa, driven by opposing underlying cellular and molecular mechanisms (Musicco et al., 2013; Shafi, 2016). In our study, 11 types of cancer fail to show significant correlations with dementia, but still, a strong inverse correlation was observed in the digestive system related to chronic inflammation, which is consistent with previous cohort studies conducted with other datasets (Kang et al., 2023). Previous epidemiological studies have widely reported an inverse association between neurodegenerative diseases and cancer, with dementia patients showing a lower cancer risk compared those without. Building upon this established inverse relationship, our retrospective study extends this perspective, revealing that patients in the pre-dementia stage also present with a lower prevalence of certain cancers. This lower cancer prevalence does not necessarily reflect an overall improvement in physical health; rather, the observed protective association may stem from aberrantly activated immune surveillance mechanisms. Further research, however, is needed to fully elucidate the underlying biological pathways.

Conversely, our study identified significant positive associations between pre-dementia status and several conditions, including OCA, brain cancer, lung cancer, prostate cancer, bladder cancer, and contusion of the lower limb. These findings are consistent with the biological plausibility that cerebral integrity is fundamentally dependent on a continuous and adequate oxygen supply. Chronic hypoxic states—whether arising from direct vascular compromise in OCA, respiratory obstruction in lung cancer, or potentially reduced systemic circulation due to sedentary behavior following lower limb contusions—may serve as a critical driver for neurodegeneration (Stewart et al., 2025; Thangwong et al., 2025). Recent evidence suggests that the severity of hypoxemia is closely correlated with the progression of cognitive dysfunction (Kakkera et al., 2018). Furthermore, abnormalities in oxygen metabolism, including chronic hypoxia and oxidative stress, have been shown to accelerate core Alzheimer’s pathologies, such as amyloid-β aggregation, tau phosphorylation, and neuroinflammation (Liu et al., 2023). While our observational design cannot establish causality, the convergence of our epidemiological findings with these mechanistic studies strengthens the biological plausibility of hypoxia-mediated pathways in pre-dementia disease associations. Similarly, direct brain pathology, as seen in brain cancer, directly contributes to cognitive decline. The positive association with bladder and prostate cancers might be explained by their common impact on sleep quality (Chung and Lin, 2019; Mogavero et al., 2021), especially the deep sleep has been documented essential for brain to be deeply cleaned via slow wave tidal brain activity (van Alphen et al., 2021). Although lower limb injuries may seem less immediate, it could reflect increased frailty, a higher risk of falls, or reduced physical activity, leading a sedentary lifestyle. all of which are associated with systemic inflammation and poorer brain health (Thangwong et al., 2025). Those findings, on the other hand, suggest a positive correlation between cancer related to brain damage, respiratory/urinary system and dementia, indicating the possible mechanisms of vascular dementia.

To avoid systematic bias, we also evaluate gout, which has been reported previously and the results remain consistently (Lu et al., 2016). Moreover, researchers may suspect that cognitive impairment of individuals with dementia might lead to a decreased screening or less reporting of symptoms (Freedman et al., 2016). This suspicion points out the importance of revealing the earlier health status of patients to exclude possible complications from medicines, and to minimize the potential for underdiagnosis among individuals with dementia. To curtail this potential bias, we analyzed the data of up to a decade prior to the diagnosis of dementia, and included multiple diseases ranging from acute infectious diseases to cancer, from PNS to CNS. Interestingly, among the 30 common diseases in this study, there is a significantly higher prevalence of 6 diseases in the pre-dementia group. One large-scale cohort study from Denmark reports that TBI, another cerebrovascular disease, seems to be associated with an increased risk of dementia (Graham and Sharp, 2023). These findings suggest a potential inverse association between pre-dementia and certain diseases.

Despite these strengths, certain limitations warrant consideration. In our study, several conditions—such as gout and skin cancer—showed a lower prevalence in the pre-dementia group compared to controls. While some literature suggests potential biological mechanisms for these inverse associations (e.g., the antioxidant properties of uric acid in gout) (Lu et al., 2016), these findings must be interpreted with caution due to several potential biases. First, underdiagnosis and detection bias may contribute to these results. Individuals in the prodromal phase of dementia may exhibit reduced healthcare-seeking behavior for conditions perceived as less urgent, leading to a lower recorded prevalence despite a similar true incidence; for example, skin cancer screening may be deprioritized as cognitive symptoms emerge. Although Taiwan’s highly accessible National Health Insurance system facilitates routine visits for mild symptoms and aids in capturing early-stage dementia, it only partially mitigates the inherent risk of under-recording. Second, differential healthcare utilization patterns may influence detection, as the pre-dementia group might allocate limited healthcare resources toward cognitive and neurological concerns at the expense of other conditions, resulting in systematically lower diagnosis rates. Finally, survival bias may artificially reduce the observed prevalence for conditions associated with increased mortality. Individuals with aggressive malignancies (e.g., pancreatic cancer) or severe chronic diseases may die before reaching the age of clinically diagnosed dementia, leading to their underrepresentation in the pre-dementia cohort.

We measured comorbid conditions prior to the recorded date of dementia diagnosis to reduce reverse causation, several limitations of temporal ascertainment remain. Diagnosis dates in our administrative data reflect first recorded encounters rather than precise clinical onset, so a fixed multi-year pre-diagnosis exclusion could introduce temporal misclassification. Moreover, despite the large overall sample, applying a 2–5 years exclusion would disproportionately reduce event counts for certain exposures and clinically important subgroups (for example, acute appendicitis, brain cancer, small-intestine cancer, and pancreas cancer), compromising precision and the stability of subgroup estimates. Retrospectively imposing a long lag would also disrupt our matched design and time-dependent covariates, necessitating substantial re-specification of models and raising additional methodological challenges. To mitigate these concerns we used a long look-back window (up to 10 years), excluded prevalent dementia cases at index, and adjusted for available proxies of healthcare utilization; nevertheless, residual influence of the prodromal phase on healthcare seeking and diagnostic coding cannot be fully excluded.

Our study lacks a protracted follow-up window after the index date for the non-demented controls. Given the long preclinical stage of dementia, it is possible that some individuals in the control group might have subsequently developed cognitive impairment. However, such misclassification would likely bias our results toward the null, implying that our observed associations are conservative and potentially underestimated. Future prospective cohort studies with extended follow-up periods are warranted to confirm these cognitive trajectories and completely rule out misclassification bias. It is necessary to conduct future prospective cohort studies with longer follow-up periods, identify the timing of symptom onset, employ more detailed clinical assessment methods, or utilize datasets capable of robust lagged sensitivity analyses, in order to further clarify the directionality of the observed associations and completely rule out misclassification bias.

Our study did not adjust for total healthcare utilization in regression models, as this may represent a mediator rather than a confounder (VanderWeele, 2019). While this approach avoids over-adjustment bias, it remains a limitation. However, our disease-specific analysis showing divergent patterns (decreased general visits but increased neurological visits) suggests associations are not solely driven by overall utilization differences. While our dataset is based exclusively on ICD diagnostic codes and outpatient visit records. We do not have access to corresponding biological samples, inflammatory markers, or immune cell profiles. Consequently, any discussion regarding a ‘hyperactive peripheral immune system’ remains strictly speculative. Future research should integrate longitudinal clinical data with detailed immunological markers, neuroimaging, and genetic analyses to comprehensively reveal the complex interactions between peripheral immunity, overall health status, and neurodegenerative processes.

Lastly, as an observational study, our findings demonstrate correlations rather than direct causality. Although the link between a reduced infectious burden and subsequent dementia suggests altered preclinical peripheral immunity, it remains unclear whether these immune shifts are causal or merely epiphenomena. Specifically, these correlations could be driven by early systemic immune alterations preceding clinical dementia. Alternatively, they may reflect reverse causality, wherein preclinical neurodegeneration compromises immune capacity, or simply arise from shared underlying confounders. Ultimately, prospective studies integrating immune biomarker profiling and experimental models are required to elucidate causality and the underlying biological mechanisms.

In conclusion, the pre-dementia cohort showed lower incidence rates of various infections and certain cancers, and required less hospital care over a decade prior to dementia diagnosis. While these findings are consistent with the hypothesis of peripheral immune involvement in AD pathogenesis, this interpretation remains speculative given the observational design and lack of mechanistic data. Conversely, conditions impairing oxygen supply or sleep quality are positively associated with future dementia risk. Our findings indicate the etiology of early dementia may correlated with the condition of the dynamic peripheral immunity, in addition to the general accepted neural causality.

## Data Availability

The raw data supporting the conclusions of this article will be made available by the authors, without undue reservation.
