# Supplementary material for: Association between pre-diagnostic prevalence of 30 common diseases and the subsequent risk of dementia: a population-based retrospective cohort study in Taiwan
Source: Front Aging Neurosci. 2026 Apr 17;18:1784183. doi: 10.3389/fnagi.2026.1784183 (PMC13133077; doi:10.3389/fnagi.2026.1784183)
Supplement: Supplementary file 1 [file Table_1.docx]

**Table S1. ICD-9 Codes Used**

| **Supplementary Table S1. ICD-9 Codes Used to Define Outcomes** | |
| --- | --- |
| Outcomes of interest | ICD-9 |
| Acute nasopharyngitis | 460 |
| Acute sinusitis | 461 |
| Acute pharyngitis | 462 |
| Acute tonsillitis | 463 |
| Acute laryngitis and tracheitis | 464 |
| Acute bronchitis and bronchiolitis | 466 |
| Gastritis and duodenitis | 535 |
| Acute appendicitis | 540 |
| Urticaria | 708 |
| Blepharitis | 373 |
| Malignant neoplasm of pancreas | 157 |
| Benign neoplasm of skin | 216 |
| Malignant neoplasm of trachea, bronchus, and lung | 162 |
| Occlusion of cerebral arteries | 434 |
| Gout | 274 |
| Senile dementia | 290 |
| Amnestic syndrome | 294 |
| Alzheimer’s disease | 331 |
| Hypertension | 401 |
| Diabetes | 250 |

The Taiwan Inpatient Registry includes mandatory information on all principal and secondary hospital discharge diagnoses. Diagnoses in the Inpatient Registry were classified according to the International Classification of Diseases (ICD) system.
